# Pseudo-beer belly disguised and presenting as inguinal hernia swelling: a case of massive retroperitoneal liposarcoma

**DOI:** 10.3389/fmed.2025.1499764

**Published:** 2025-05-02

**Authors:** Junfeng Xie, Kexing Xi, Baolong Ye, Yangbiao Wang, Hongquan Liu, Hefang Xiao

**Affiliations:** ^1^Department of Gastrointestinal Hernia Surgery, Ganzhou Hospital-Nanfang Hospital, Southern Medical University, Ganzhou, Jiangxi, China; ^2^Cancer Center, Beijing Tsinghua Changgung Hospital, School of Clinical Medicine, Tsinghua University, Beijing, China; ^3^Department of Emergency, The First Affiliated Hospital of Nanchang University, Nanchang, Jiangxi, China

**Keywords:** retroperitoneal liposarcoma, inguinal hernia, MDM2 amplification, surgical resection, surveillance

## Abstract

**Background:**

Liposarcoma, a rare malignancy accounting for 1% of all cancers, constitutes 9.8–16% of soft tissue sarcomas. The retroperitoneum and extremities are primary sites of occurrence. Retroperitoneal liposarcoma (RLS), originating in retroperitoneal adipose tissue near the kidneys and mesentery, represents 0.07–0.2% of all tumors. Giant RLS is uncommon, and its presentation via inguinal herniation is exceedingly rare.

**Case description:**

A 43-year-old male presented with progressive right inguinal swelling over one year, initially diagnosed as an irreducible inguinal hernia. Physical examination revealed a non-pulsatile, soft inguinal mass persisting in supine position. Due to the patient’s abdominal obesity and atypical resilience of the mass, abdominal computed tomography (CT) was performed, identifying a large fatty lesion (25 × 22 × 32 cm) in the right abdomen and pelvis with septations and dense areas. Exploratory laparotomy revealed a lobulated, encapsulated retroperitoneal tumor adherent to the right kidney, retroperitoneal vasculature, and left ureter, with a nodule extending into the inguinal canal. En bloc resection and hernia repair were performed. Histopathology confirmed well-differentiated liposarcoma (WDLS) with MDM2/CDK4 overexpression and MDM2 amplification via fluorescence *in situ* hybridization (FISH). No recurrence was observed during follow-up (3–12 months postoperatively).

**Conclusion:**

In obese patients with abdominal obesity and irreducible inguinal herniation lacking obstructive symptoms, retroperitoneal tumors should be considered. Imaging (CT/MRI) and molecular testing (MDM2 FISH) are critical for differential diagnosis. Complete surgical excision with safe margins remains the cornerstone of management, followed by rigorous surveillance.

## Introduction

Soft tissue sarcomas are rare tumors, accounting for 1% of all malignancies. Liposarcomas comprise 9.8–16% of soft tissue sarcomas. The two main sites where liposarcomas tend to occur are the extremities and the retroperitoneum. Retroperitoneal liposarcoma is the most common primary retroperitoneal malignant tumor, which can originate from normal adipose tissue in the retroperitoneal space, and is more frequently found around the kidneys and within the mesentery, accounting for 0.07–0.2% of all tumors. The average age of onset is between 40 and 60 years, and the incidence is similar in both genders (1). A giant liposarcoma is typically defined as a tumor with a diameter of 30 cm or more or a weight of 20 kg or more (2). Retroperitoneal giant liposarcoma is a relatively rare tumor, and it is extremely uncommon for a retroperitoneal sarcoma to protrude through the inguinal region. Herein, a rare case of a retroperitoneal giant liposarcoma masquerading as a beer belly and presenting as inguinal hernia swelling is reported, along with a discussion of the difficulties in preoperative diagnosis and the extent of surgery.

## Case report

**Figure 1:**
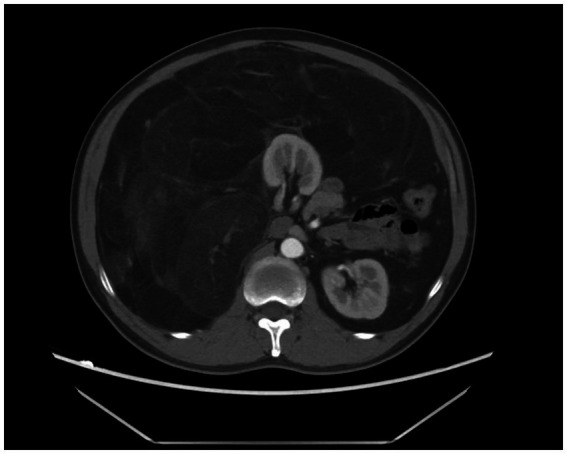
Abdominal CT showing a large fat-density mass with septations and hyperdense areas in the right retroperitoneal/pelvic region.

**Figure 2:**
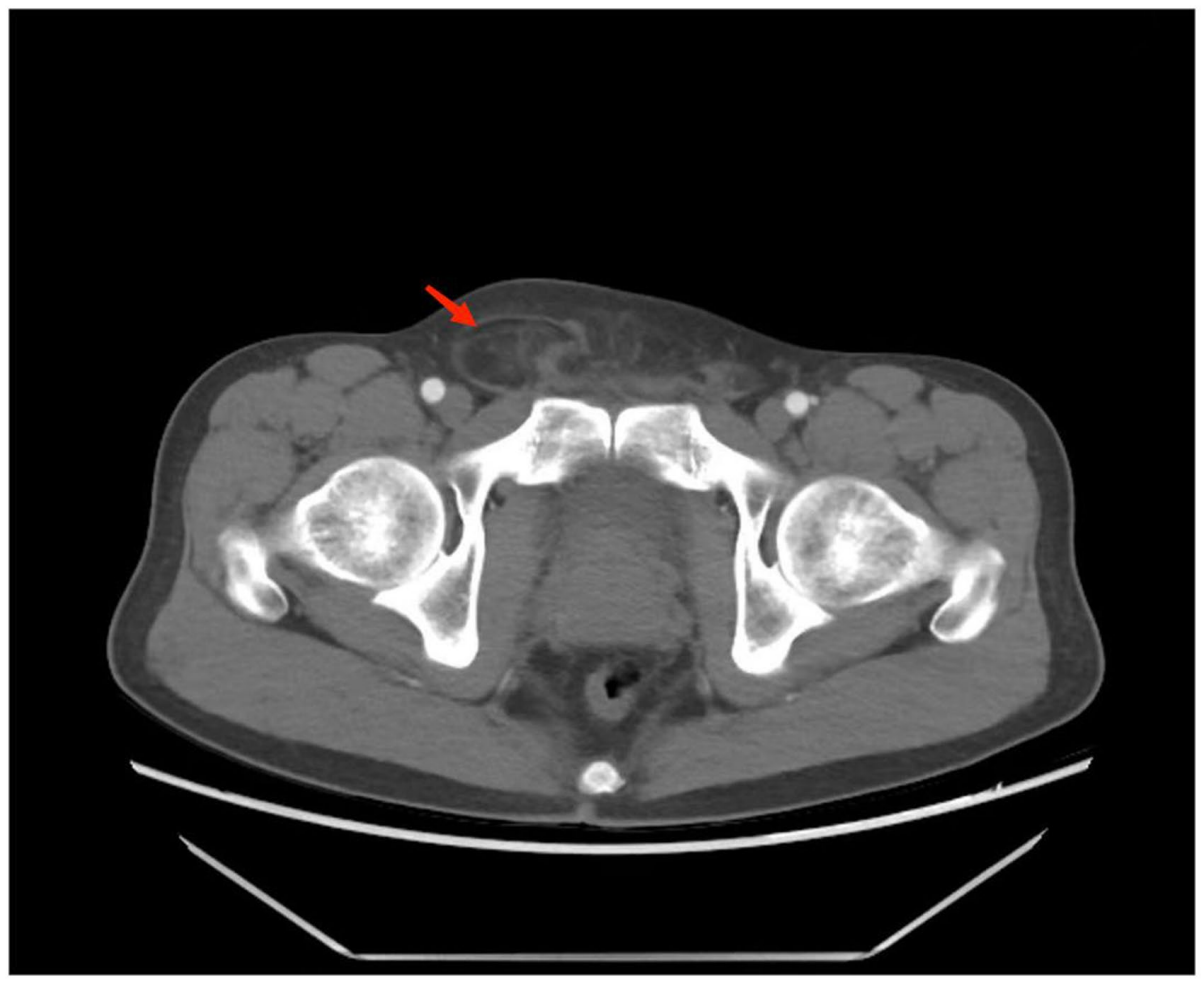
Herniation of the tumor into the inguinal canal (arrow).

A 43-year-old male patient was admitted to our hospital. The patient presented with swelling in the right inguinal region that had been present for one year, and the discomfort in the inguinal area gradually worsened over the next six months, but without symptoms such as nausea, vomiting, or constipation. He was diagnosed with a right inguinal hernia and was hospitalized. His blood test results were normal. Physical examination revealed a non-reducible swelling in the right inguinal region in the standing position, while the other side was normal. The swelling in the right inguinal region was also difficult to reduce in the supine position. The inguinal bulge was soft, without a pulse. However, the patient was overweight, with a distended abdomen resembling a beer belly, and it felt resilient upon palpation. Considering the possibility of an associated abdominal lesion, the patient was advised to undergo an abdominal CT scan. The CT scan showed a huge adipose density mass in the right side of the abdominal and pelvic cavities, with multiple septal and flocculent density-enhanced shadows within. The lower edge of the mass was seen to extend into the inguinal region, with a size of approximately 25 × 22 × 32 cm. After contrast enhancement, the septal and flocculent density-enhanced shadows showed mild enhancement. The right kidney, pancreas, and the intestinal tract in the abdominal and pelvic cavities were compressed and displaced to the left (Figures 1, 2).

**Figure 3:**
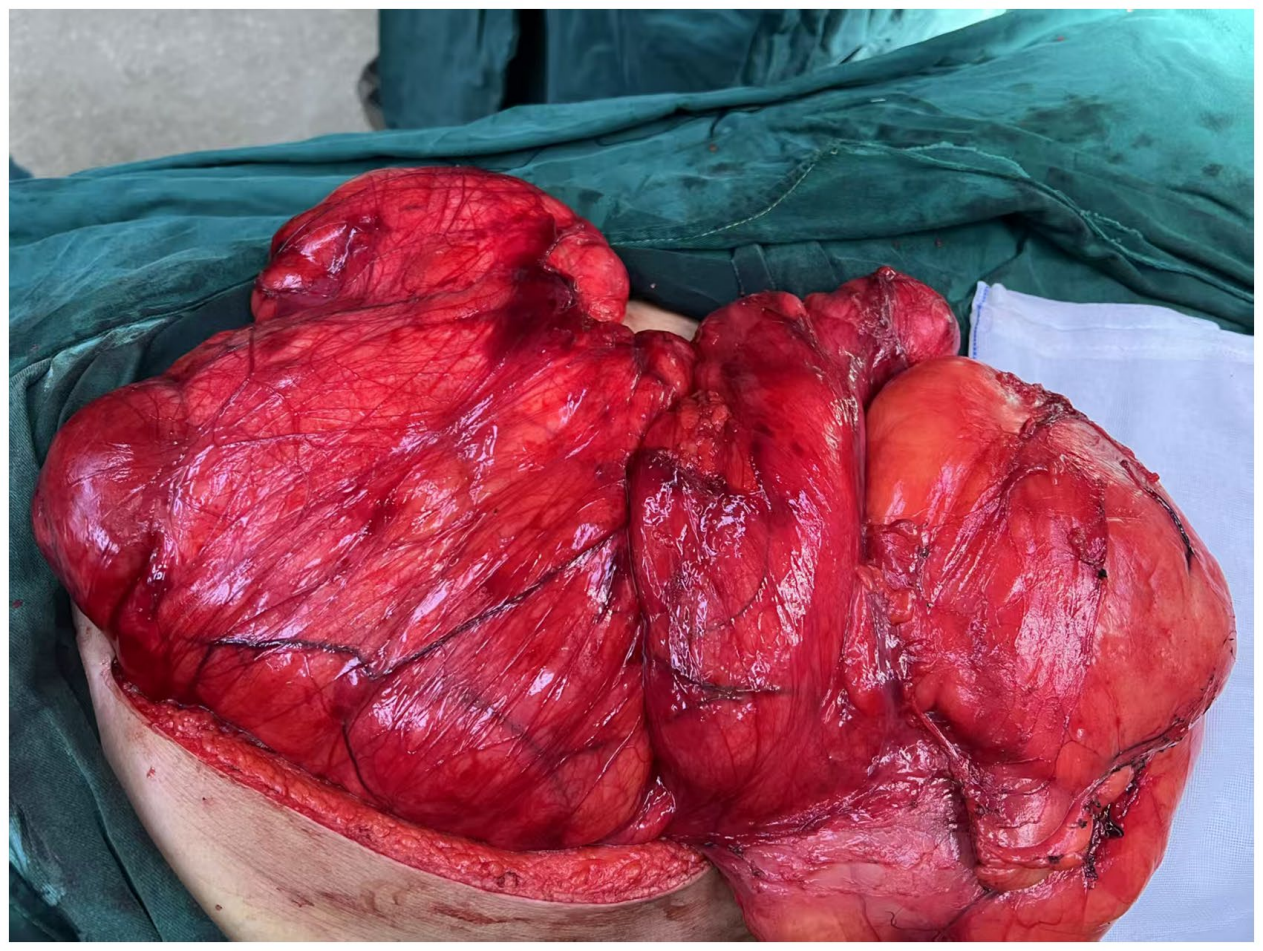
Intraoperative view of the lobulated, encapsulated retroperitoneal mass.

**Figure 4:**
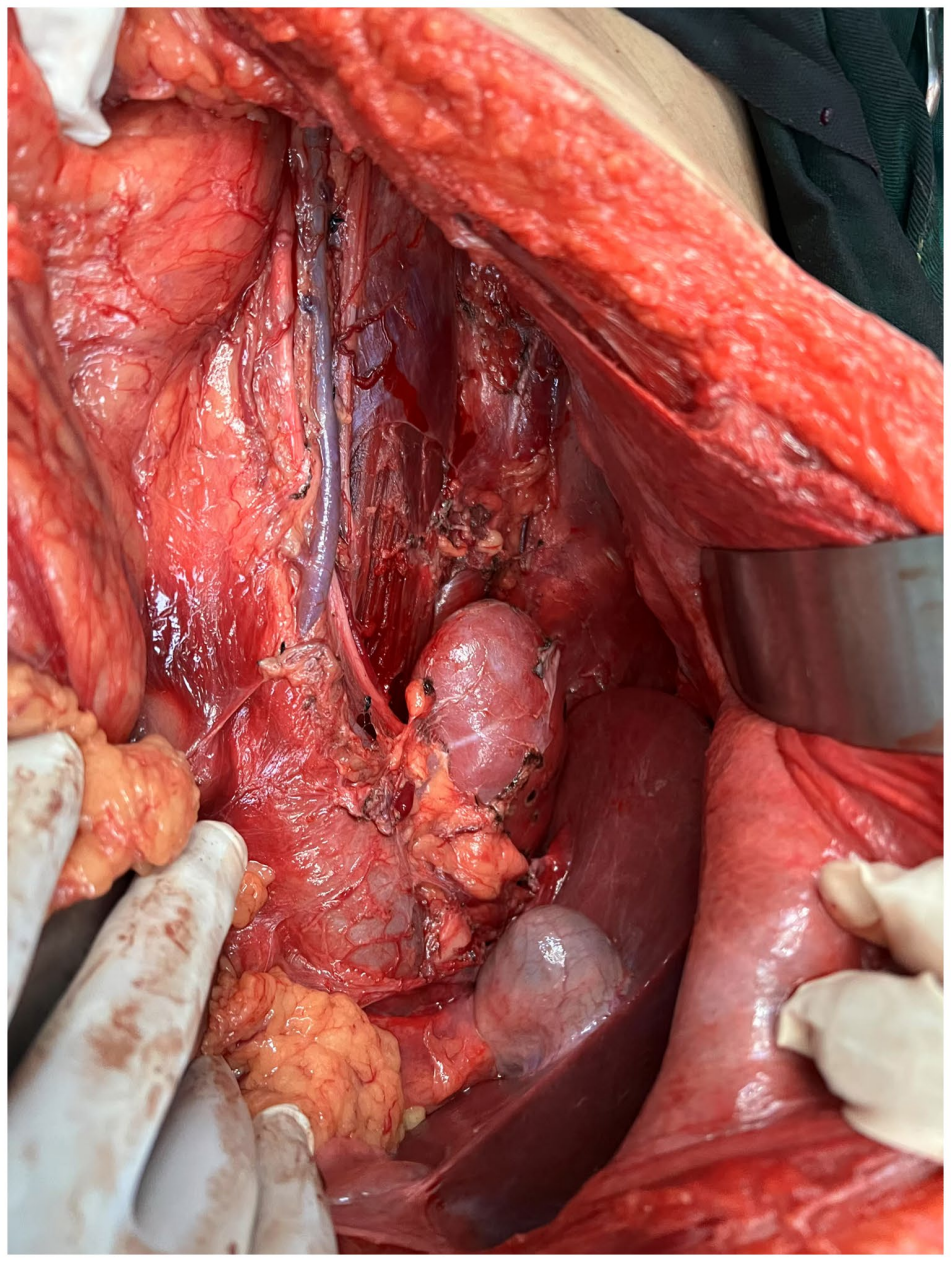
Tumor adhesions to retroperitoneal vessels, kidney, and ureter.

After completing all relevant examinations and finding no surgical contraindications, the patient underwent laparotomy on the third day of admission. During the operation, the tumor was found to be located in the retroperitoneum and pelvic cavity. The surface of the tumor was lobulated and globular, with a complete capsule. Its base was extensively adhered to retroperitoneal major blood vessels, the right kidney, the right spermatic vessels, the left ureter, and other organs and structures. One nodule of the mass dropped down to form an inguinal hernia (Figures 3, 4). After reducing the hernia, the entire huge mass was excised, and the defect in the inguinal region was repaired.

**Figure 5:**
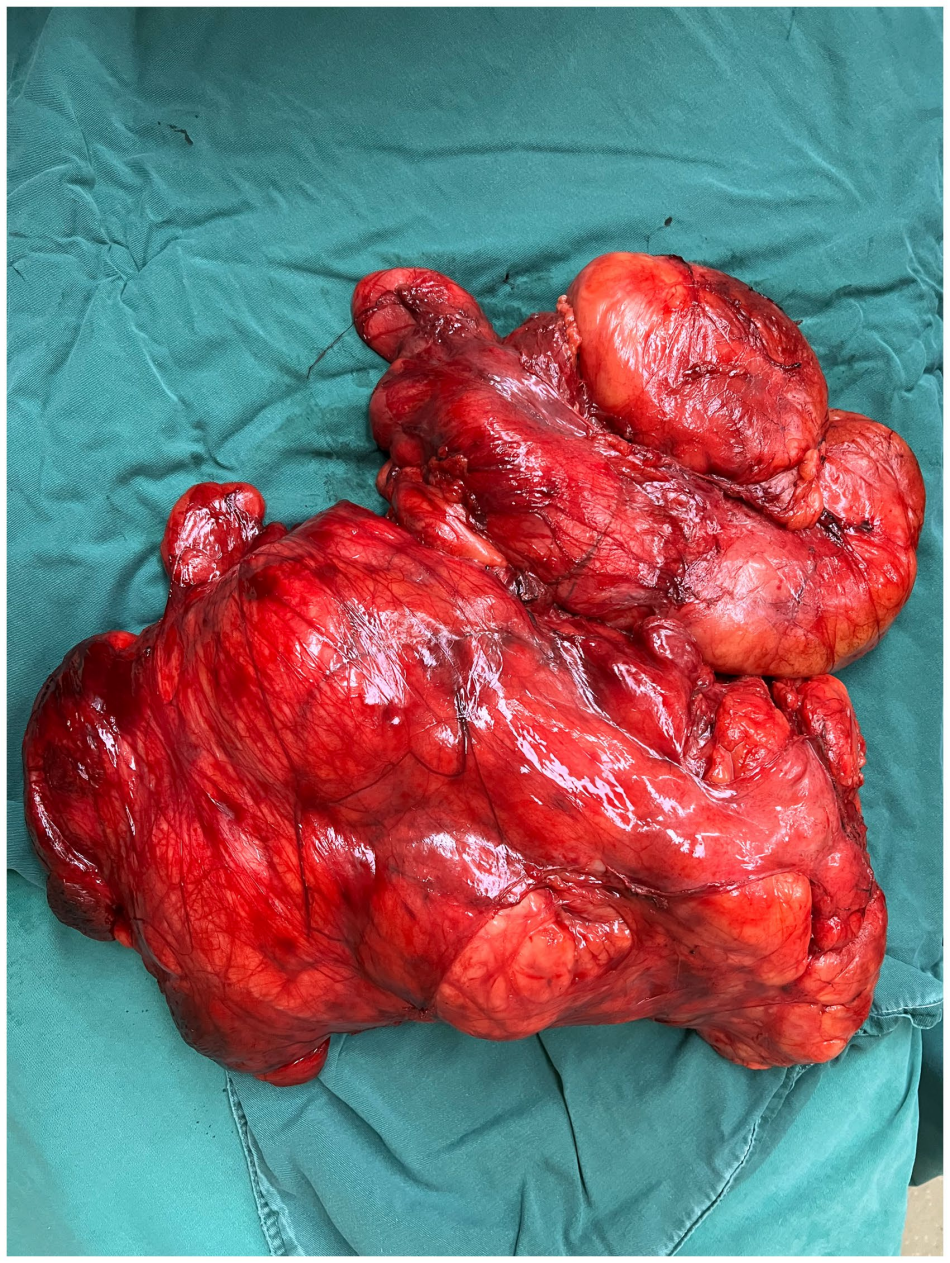
Gross specimen with fibrous septations and gray-yellow cut surface.

On the fourth day after the operation, the pathological results of the patient were reported from the pathology laboratory. The mass was approximately 36 × 30 × 26 cm in size, and the cut surface was gray-yellow and gray-white (Figure 5).

**Figure 6:**
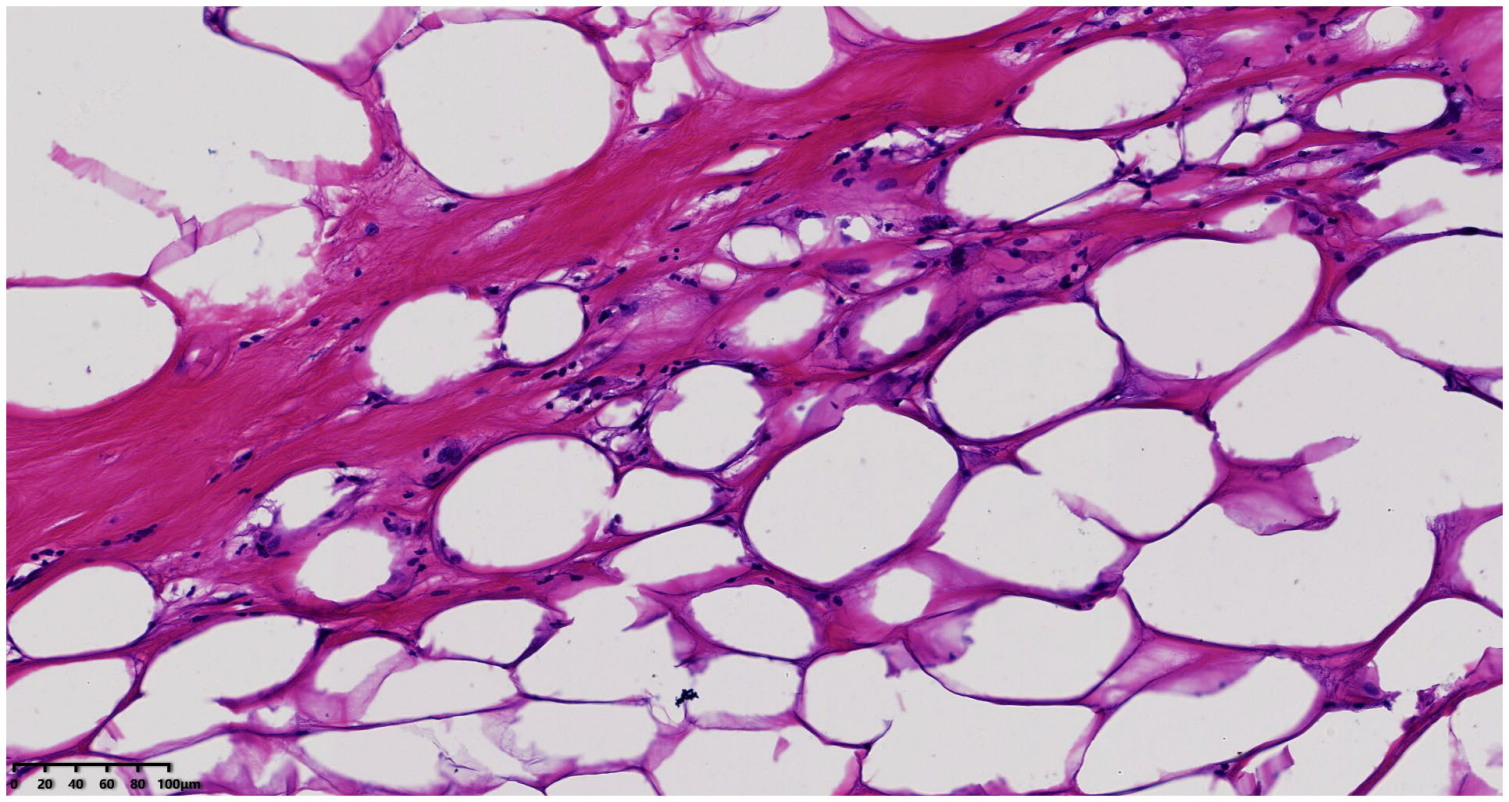
Histopathology (H&E, ×200) showing dense fibrosis and hyperchromatic cells.

**Figure 7:**
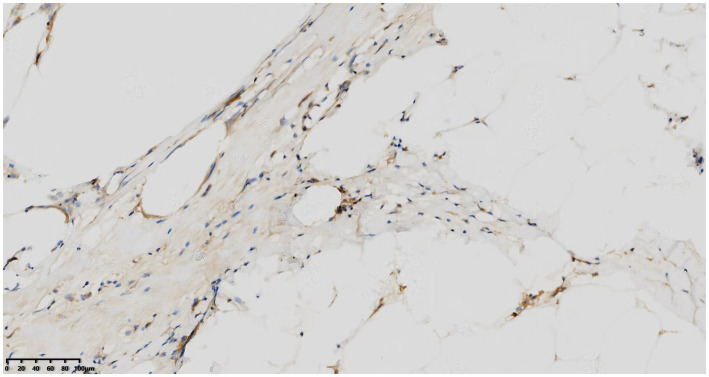
Immunohistochemical nuclear positivity for MDM2.

**Figure 8:**
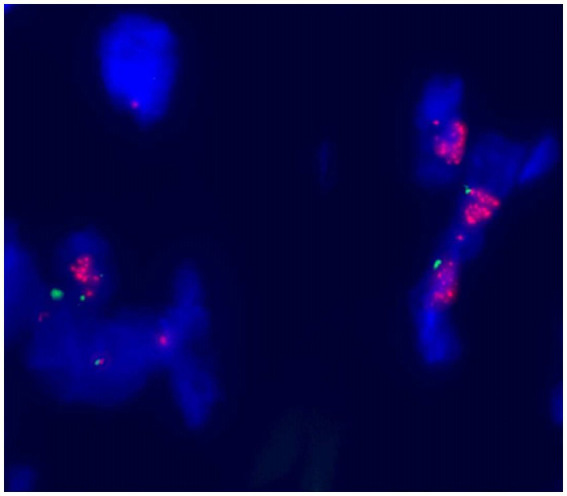
FISH analysis demonstrating MDM2 amplification (red/green ratio > 2).

Histological examination showed that the tumor contained large areas of dense collagen fibrosis, and irregular cells with deeply stained nuclei were visible within the fibrotic areas (Figure 6). Immunohistochemical analysis revealed that vimentin (Vim), SOX-10, S-100, P53, p16, Ki67, CDK4, and MDM2 (Figure 7) were positive in the nucleus. Fluorescence *in situ* hybridization (FISH) analysis showed that the red signals were distributed in small clusters under the microscope, and the red/green signal ratio was greater than 2 (Ratio value >2), indicating positive MDM2 gene amplification (Figure 8). The final diagnosis was well-differentiated liposarcoma.

On the fifth day after the operation, the patient recovered well, and all examination indicators were normal. Thus, the patient was discharged smoothly. The patient did not receive any other treatment after discharge. The patient was followed up at 3 months, 6 months, and 12 months after the operation. Abdominal CT scans were all normal, showing no signs of recurrence.

## Discussion

Liposarcomas are classified into five types according to their molecular and cytological characteristics and immunohistochemical features: well-differentiated, dedifferentiated, myxoid or round cell type, pleomorphic, and mixed types. The well-differentiated and dedifferentiated subtypes are more common in the retroperitoneal cavity (3). Their clinical behavior lies between that of well-differentiated liposarcomas and high-grade sarcomas. The local recurrence rate is 41%, the metastasis rate is 17%, and the disease-related mortality rate is 28%. Prognosis is mainly affected by local recurrence, especially in the retroperitoneal region (4, 5). Retroperitoneal liposarcomas have a large growth space, and since this tumor grows expansively and generally does not infiltrate, clinical symptoms appear late, making early detection difficult. Symptoms only occur when the tumor is large, adheres to, compresses, or affects adjacent organs, especially when it displaces, compresses, or encircles the abdominal aorta, inferior vena cava, and iliac vessels that run through the retroperitoneal space (6). The retroperitoneal cavity communicates with the inguinal region through cord-like structures. Therefore, when the mass is huge, part of the tumor tissue can drop down to form an inguinal hernia. Whether the hernia content can be reduced or not, the patient usually has no abdominal pain or intestinal obstruction symptoms (7). In our case, due to the patient’s obesity, the bulging abdomen was mistaken for a beer belly, and the presence of the retroperitoneal liposarcoma was not detected until an inguinal hernia formed.

Computed tomography (CT) is the preferred imaging method for diagnosing retroperitoneal liposarcoma (8). Combining the patient’s medical history, symptoms, signs, physical examination, and imaging examinations (such as B-ultrasound, CT, and MRI) can generally lead to a diagnosis. However, the gold standard for diagnosis remains live tissue pathological examination and postoperative pathological histological examination. Differentiating between lipomas and well-differentiated liposarcomas is a diagnostic challenge. CT imaging features suggesting malignancy include a large lesion area, the presence of thick septa, the presence of nodules and/or globular or non-fatty masses, and a reduced percentage of fat components (9). Histopathological examination is the basis for diagnosing lipomas, showing fibrous septa containing enlarged, deeply stained cells and rare lipoblasts (10). However, some well-differentiated liposarcomas with adipose differentiation may be extremely difficult to distinguish from lipomas due to the presence of very few deeply stained cells and lipoblasts. In such cases, an immunohistochemical method consisting of MDM2 and CDK4 is recommended. Fluorescence *in situ* hybridization (FISH) for MDM2 amplification can be used to distinguish between lipomas, atypical lipomas, and well-differentiated liposarcomas (11). In our case, both MDM2 and CDK4 were positive.

The treatment principle for retroperitoneal liposarcoma is to completely excise the tumor within the scope of surgical indications and under the premise of ensuring safety, while meeting the safety margin criteria. For advanced patients with unresectable locally recurrent liposarcomas or distant metastatic high-grade liposarcomas, chemotherapy and radiotherapy can be combined (12–14). During the operation, it was found that in addition to the difficulty in identifying and preserving retroperitoneal organs and structures, the highly differentiated fat cells in liposarcomas made it difficult to distinguish them from normal retroperitoneal fat. Therefore, determining the safe resection margin became challenging. Based on our surgical experience and the interpretation of relevant literature (9, 15, 16), it is considered that during surgical resection, the resection should be as far away from the visible boundary of the tumor as possible, and the false capsule of the tumor should be completely excised. If necessary, combined organ resection may be required to ensure complete tumor resection. Extended resection can reduce the postoperative recurrence rate of well-differentiated liposarcomas. After completely excising the tumor, the surrounding suspicious adipose tissue can be further removed. Among them, combined organ resection mainly involves the kidney. However, major organs should be preserved as much as possible. It should be noted that organ compression does not necessarily mean organ invasion. During the operation, some tissues should be appropriately selected for frozen pathological examination to prevent blind expansion of the resection range. Some studies (17, 18) have shown that for patients with multiple recurrences, combined organ resection can increase the thoroughness of resection. Common organs to be resected include the kidney, spleen, colon, duodenum, etc. The prognosis of patients after radical resection is significantly better than that of those with partial resection or palliative resection. Some patients may find it difficult to achieve radical tumor resection due to factors such as large tumor size, complex adjacent relationships, and invasive growth of the tumor. In such cases, palliative resection can be chosen to relieve compression and obstruction and alleviate the patient’s symptoms. In our case, the huge tumor was closely related to the left ureter. Therefore, a left ureteral stent was placed before the operation to avoid accidental injury to the left ureter during the operation. Since the visible boundary of the tumor was at a safe distance from important organs and structures, the tumor was relatively completely excised, and the surrounding suspicious adipose tissue was removed. Some tissues were selected for frozen pathological examination during the operation, and the examination results indicated negative resection margins. Therefore, an extended operation was not performed. It is worth noting that during the operation, it was difficult to distinguish the boundary between the tumor tissue and the normal perirenal adipose tissue. To ensure a relatively safe surgical margin, all perirenal adipose tissue was resected while the kidney was preserved.

The Transatlantic Australasian Retroperitoneal Sarcoma Working Group (TARPSWG), composed of clinicians from more than 35 sarcoma centers in Europe and North America, recommends that for histological types with a relatively slow clinical course, such as well-differentiated liposarcoma, CT imaging surveillance should be performed every 3–6 months for 3 years, and then annually (19).

## Conclusion

In obese patients presenting with abdominal protrusion resembling a beer belly, the diagnostic evaluation of irreducible inguinal hernias should prioritize consideration of abdominal neoplasms when accompanied by absent symptoms of abdominal pain or intestinal obstruction. Under such clinical circumstances, comprehensive abdominal and pelvic imaging modalities—including color Doppler ultrasound, computed tomography (CT), or magnetic resonance imaging (MRI)—should be promptly performed to minimize diagnostic oversight. Contrast-enhanced CT scans are particularly valuable for assessing vascular invasion, distant metastasis, and spatial relationships between tumors and adjacent vasculature. Concurrently, MRI provides superior soft tissue characterization, enabling detailed analysis of lesion composition.

The combination of imaging examinations and molecular detection facilitates the differential diagnosis of retroperitoneal liposarcoma versus lipoma. In cases meeting surgical criteria, complete tumor excision with adequate safety margins is recommended when technically feasible, followed by rigorous postoperative surveillance.

This investigation is constrained by its single-case design and limited follow-up duration. Multicenter studies with extended observation periods are warranted to corroborate these clinical observations and enhance the generalizability of the proposed diagnostic protocol.

## Data Availability

The original contributions presented in the study are included in the article/supplementary material, further inquiries can be directed to the corresponding authors.

## References

[1] Bachmann R Eckert F Gelfert D Strohäker J Beltzer C Ladurner R. Perioperative strategy and outcome in giant retroperitoneal dedifferentiated liposarcoma-results of a retrospective cohort study. World J Surg Oncol. (2020) 18:296. doi: 10.1186/s12957-020-02069-2, PMID: 33183309 PMC7664077

[2] Bibi M Ben Rhouma S Ouanes Y Chelly B Ghorbel Z Sellami A. Fatty tumors of the retroperitoneum: lipoma or well-differentiated liposarcoma. About a case of a giant retroperitoneal liposarcoma. Urol Case Rep. (2018) 21:58–60. doi: 10.1016/j.eucr.2018.08.020, PMID: 30211007 PMC6134184

[3] Dehner CA Hagemann IS Chrisinger JSA. Retroperitoneal Dedifferentiated Liposarcoma. Am J Clin Pathol. (2021) 156:920–5. doi: 10.1093/ajcp/aqab051, PMID: 34125170

[4] Sengul I Sengul D. Deep soft tissue leiomyoma of the lower extremities: a case report. Acta Chir Belg. (2009) 109:112–3. doi: 10.1080/00015458.2009.11680386, PMID: 19341211

[5] Sengul D Sengul I Ustun H. Dedifferentiated Liposarcoma of the left thigh: a rare case. Med Arch. (2019) 73:121–2. doi: 10.5455/medarh.2019.73.121.122, PMID: 31391701 PMC6643339

[6] Rives-Lange C Poghosyan T Dariane C Douard R Mongeois E Boudaoud AA. Delayed retroperitoneal liposarcoma diagnosis and management in a patient with massive obesity. Eur J Clin Nutr. (2021) 75:1520–2. doi: 10.1038/s41430-020-00855-5, PMID: 33649526

[7] Lechner M Borhanian K Mitterwallner S Bittner R Klieser E Köhler G. Retroperitoneal Liposarcoma: a concern in inguinal hernia repair. JSLS. (2019) 23:e2018.00064. doi: 10.4293/JSLS.2018.00064, PMID: 30700965 PMC6345196

[8] Matthyssens LE Creytens D Ceelen WP. Retroperitoneal liposarcoma: current insights in diagnosis and treatment. Front Surg. (2015) 2:4. doi: 10.3389/fsurg.2015.00004, PMID: 25713799 PMC4322543

[9] Chen J Hang Y Gao Q Huang X. Surgical diagnosis and treatment of primary retroperitoneal Liposarcoma. Front Surg. (2021) 8:672669. doi: 10.3389/fsurg.2021.672669, PMID: 34150840 PMC8211986

[10] Chrisinger JSA Al-Zaid T Keung EZ Leung C Lin HY Roland CL. The degree of sclerosis is associated with prognosis in well-differentiated liposarcoma of the retroperitoneum. J Surg Oncol. (2019) 120:382–8. doi: 10.1002/jso.25585, PMID: 31206726 PMC7652035

[11] Tyler R Dilworth MP James J Blakeway D Stockton JD Morton DG. The molecular landscape of well differentiated retroperitoneal liposarcoma. J Pathol. (2021) 255:132–40. doi: 10.1002/path.5749, PMID: 34156092

[12] Casadei L De Faria FCC Lopez-Aguiar A Pollock RE Grignol V. Targetable pathways in the treatment of retroperitoneal Liposarcoma. Cancers. (2022) 14:1362. doi: 10.3390/cancers14061362, PMID: 35326514 PMC8946646

[13] Littau MJ Kulshrestha S Bunn C Agnew S Sweigert P Luchette FA. The importance of the margin of resection and radiotherapy in retroperitoneal liposarcoma. Am J Surg. (2021) 221:554–60. doi: 10.1016/j.amjsurg.2020.11.041, PMID: 33256943 PMC7987801

[14] Littau MJ Bunn C Kim P Kulshrestha S Tonelli C Abdelsattar ZM. Low and moderate grade retroperitoneal liposarcoma: is adjuvant radiotherapy associated with improved survival in patients undergoing R1 resection? Am J Surg. (2022) 223:527–30. doi: 10.1016/j.amjsurg.2021.12.026, PMID: 34974888

[15] Teixeira FR Jr Arakaki MS Lima HVG de Oliveira Ferreira F Menegozzo CAM Silva ER. Multivisceral resection for retroperitoneal liposarcoma-is it worth it? A 20-year single-center experience. Surg Today. (2023) 53:1181–7. doi: 10.1007/s00595-023-02731-8, PMID: 37606758

[16] Xiao J Liu J Chen M Liu W He X. Diagnosis and prognosis of retroperitoneal Liposarcoma: a single Asian center cohort of 57 cases. J Oncol. (2021) 2021:1–10. doi: 10.1155/2021/7594027, PMID: 34035812 PMC8116140

[17] Liao T Du W Li X He S Guan G Zhu H. Recurrent metastatic retroperitoneal dedifferentiated liposarcoma: a case report and literature review. BMC Urol. (2023) 23:63. doi: 10.1186/s12894-023-01252-3, PMID: 37095466 PMC10123999

[18] Masaki N Onozawa M Inoue T Kurobe M Kawai K Miyazaki J. Clinical features of multiply recurrent retroperitoneal liposarcoma: a single-center experience. Asian J Surg. (2021) 44:380–5. doi: 10.1016/j.asjsur.2020.10.015, PMID: 33191070

[19] Zaidi MY Canter R Cardona K. Post-operative surveillance in retroperitoneal soft tissue sarcoma: the importance of tumor histology in guiding strategy. J Surg Oncol. (2018) 117:99–104. doi: 10.1002/jso.24927, PMID: 29193081

